# Primary CNS Lymphoma Arising from the 4^th^ Ventricle: A Case Report and Review of the Literature

**DOI:** 10.1155/2019/2671794

**Published:** 2019-04-10

**Authors:** Ava Brozovich, Donald Ewing, Ethan Burns, Courtney Hatcher, Gonzalo Acosta, Usman Khan, Betty Chung, Leena Samuel, Jasleen Randhawa, Sai Ravi Pingali

**Affiliations:** ^1^Texas A&M University College of Medicine, 8441 Riverside Parkway, Bryan, TX 77807, USA; ^2^Houston Methodist Hospital, Department of Medicine, 6550 Fannin St., Houston, TX 77030, USA; ^3^Houston Methodist Hospital, Department of Pathology and Genomic Medicine, 6550 Fannin St., Houston, TX 77030, USA

## Abstract

A 65-year-old male with a history of ischemic strokes, seizures, and subarachnoid hemorrhage presented with a 4-week history of progressive diplopia, vertigo, nausea, and vomiting. Magnetic resonance imaging (MRI) revealed a 2.5 × 1.8 × 1.7 cm posterior fossa mass arising from the roof of the 4^th^ ventricle extending into the cerebellar vermis. Posterior fossa craniotomy with stereotactic biopsy confirmed a locally invasive diffuse large B-cell lymphoma (DLBCL). Primary central nervous system lymphoma (PCNSL) arising from the 4^th^ ventricle is a rare extranodal manifestation of non-Hodgkin lymphoma (NHL), with few cases documented in the literature. Review of available cases lends support that lymphoma arising from the 4^th^ ventricle has a variable clinical presentation, occurs most commonly in immunocompetent males, and should be on the differential of any immunocompetent adult presenting with a posterior fossa mass. Optimal treatment modalities are based largely on phase 2 clinical trials, and recommended guidelines regardless of anatomic location should be adhered to.

## 1. Introduction

PCNSL is a rare form of extranodal NHL comprising 2.7-4.0% of central nervous system (CNS) tumors [1, 2], with an age-adjusted incidence rate of 4 cases per million persons per year [3]. PCNSL originates in the brain, leptomeninges, spinal cord, or eyes [4] and is morphologically indistinguishable from other sites of extranodal NHL [5]. The most common subtype is DLBCL, accounting for approximately 90% of PCNSLs [6]. While PCNSL is an infrequent diagnosis, rarer still is the diagnosis of intraventricular lymphoma, with a limited number of cases to contribute to the knowledge of clinical manifestations, diagnostic modalities, and optimal treatment [7–22]. The following case adds to the limited clinical knowledge of 4^th^ ventricular PCNSL.

## 2. Case

**Figure 1:**
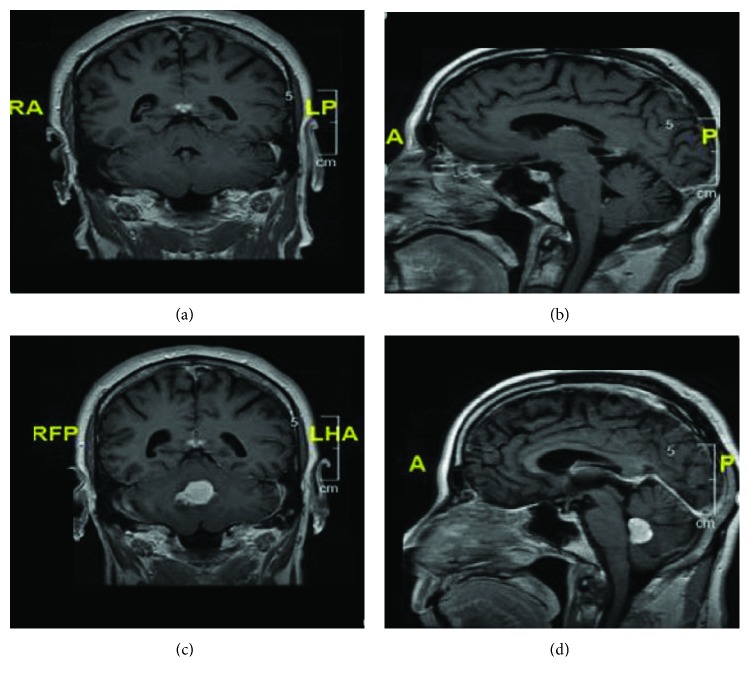
(a) Coronal T1 flair, postcontrast imaging 2 months prior to admission. No enhancing lesion seen. (b) Sagittal T1 flair, postcontrast imaging 2 months prior to admission. No enhancing lesion seen. (c) Coronal T1 flair, postcontrast imaging. There is a 1.7 × 2.5 × 1.8 cm homogenously enhancing mass with mild perilesional edema. (d) Sagittal T1 flair, postcontrast imaging. There is a 1.7 × 2.5 × 1.8 cm homogenously enhancing mass arising from the roof of the 4^th^ ventricle invading into the cerebellar vermis.

A 65-year-old Caucasian male with a pertinent history of ischemic stroke, subarachnoid hemorrhage, and recent onset of simple partial seizures 2 months prior to admission presented with a 4 week history of worsening diplopia, vertigo, nausea, and vomiting. These symptoms were initially intermittent but had become unremitting during his initial presentation. The patient denied focal neurologic deficits, ataxia, hallucinations, headaches, fevers, chills, or night sweats. The patient underwent an MRI and magnetic resonance venography (MRV) upon seizure onset that revealed 2 areas of chronic hemorrhage but was otherwise unremarkable (Figure 1).

On admission, vital signs were stable. Physical exam demonstrated rightward horizontal nystagmus, 20/40 visual acuity bilaterally, and subtle bilateral dysmetria on finger-to-nose test. A complete neurologic exam was otherwise normal. Labs were unremarkable.

**Figure 2:**
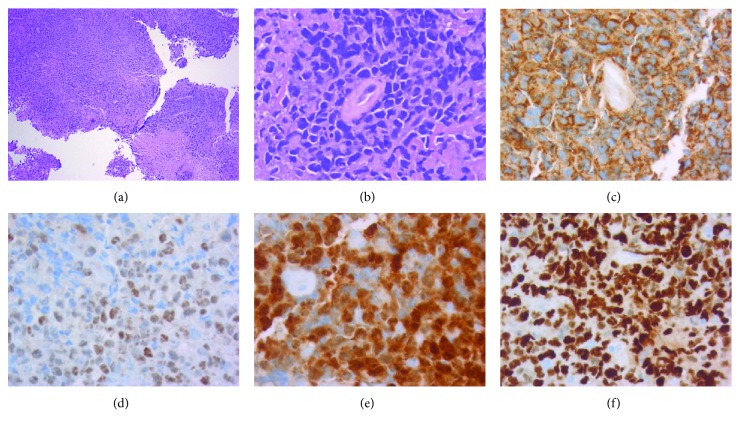
Primary CNS lymphoma arising from the 4^th^ ventricle. (a) Diffuse sheets of lymphoid tumor cells with focal necrosis, H&E stain, 40x magnification. (b) Large atypical tumor cells with angiocentric localization, H&E stain, 400x magnification. (c) CD20 immunostain, 400x magnification. (d) BCL-6 immunostain, 400x magnification. (e) MUM-1 immunostain, 400x magnification. (f) Ki-67 (MIB-1) immunostain (80% proliferation index), 400x magnification.

**Figure 3:**
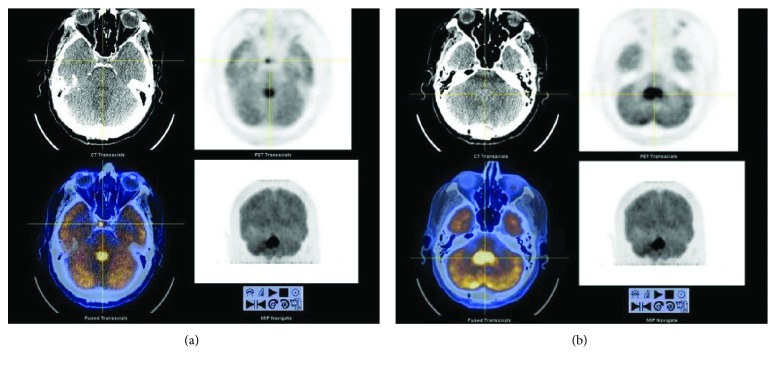
PET imaging of the brain showing increased uptake in the pituitary (a) and the posterior fossa (b). PET: positron emission tomography.

An MRI showed a 2.5 × 1.8 × 1.7 cm homogenously enhancing mass that extended from the roof of the 4^th^ ventricle (Figure 1). Perilesional edema was present without mass effect or obstructive hydrocephalus. The patient was started on dexamethasone and underwent a posterior fossa craniotomy with stereotactic biopsy that showed locally invasive disease extending from the roof of the 4^th^ ventricle into the cerebellar vermis. Intraoperative frozen sectioning revealed sheet-like arrangements of highly pleomorphic lymphoid tumor cells with atypical mitotic figures and focal necrosis, suggestive of lymphoma. Permanent sections confirmed the findings and highlighted the diffuse and angiocentric nature of the lymphoma, which was comprised primarily of large-sized lymphoma cells (Figure 2). Relevant immunohistochemical staining was positive for CD45, CD20, CD79a, MUM-1, MIB-1 (Ki-67: 80% proliferation rate), Bcl-6, and Bcl-2 and negative for CD3, CD5, CD10, CD30, C-MYC, and EBER in situ hybridization. The final histopathologic diagnosis was DLBCL with a postgerminal center phenotype. The patient had peripheral blood flow cytometry with 1% clonal B cells coexpressing CD5 with surface kappa light chain restriction, possibly representing a monoclonal B cell lymphocytosis. Cerebrospinal fluid (CSF) flow cytometry was negative for malignancy. Lactate dehydrogenase (LDH) was within normal limits. Positron emission tomography (PET) indicated increased uptake (SUV of 19.3) in the 4^th^ ventricular mass as well as a small focus of uptake in the right pituitary gland (Figure 3). Staging workup with computed tomography (CT) of the chest, abdomen, and pelvis, as well as whole body PET scan, was otherwise negative for metastasis.

The patient was initiated on rituximab, methotrexate, and cytarabine, followed by intrathecal methotrexate and a combination of cyclophosphamide, vincristine, doxorubicin, and dexamethasone (Hyper-CVAD), with plans for subsequent treatment with temozolomide and whole-brain radiation. After receiving his first dose of rituximab and methotrexate, he noted significant improvement in his symptoms. After his second cycle of Hyper-CVAD, repeat imaging showed resolution of the masses; he has been on single agent ibrutinib as maintenance therapy since and without recurrence for 10 months.

## 3. Discussion

**Figure 4:**
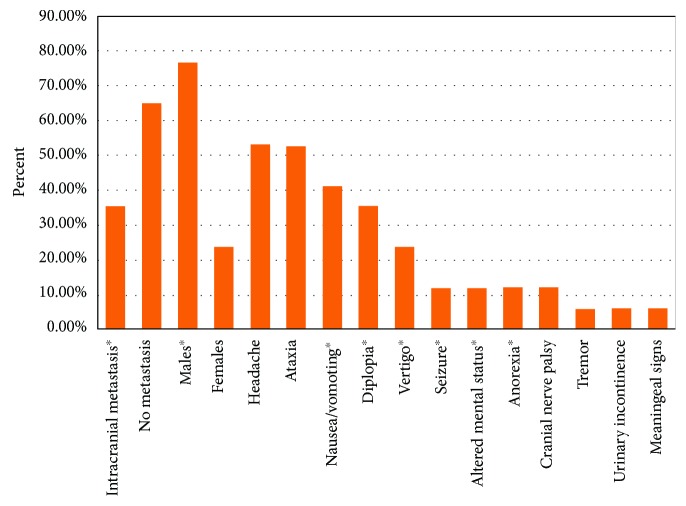
Characteristics of patients with 4^th^ ventricular PCNSL on initial diagnosis. PCNSL: primary central nervous system lymphoma.

In general, patients with PCNSL develop neurologic symptoms over the course of weeks to months. The symptoms depend on the site of involvement but can include focal neurologic deficits (56-70%), altered mental status (32-43%), symptoms related to elevated intracranial pressure (headache, nausea, and vomiting) (32-33%), and seizures (11-14%) [23, 24]. Imaging typically reveals a single brain lesion (66%), commonly in the supratentorial region (87%) [23]. Literature review of patients with 4^th^ ventricular PCNSL indicates all patients were immunocompetent, had a median age of onset at 61-years-old, and had a male-to-female predominance (3.25:1) and intracranial metastatic disease in 35.3% of cases, with 50% of these documenting metastases to other ventricles (Table 1, Figure 4) [7–22].

PCNSL should be considered in any adult presenting with a 4^th^ ventricular mass. Imaging may allude towards a PCNSL. CT may demonstrate hyper or isoattenuated lesions with marked contrast enhancement in immunocompetent PCNSL, whereas MRI may demonstrate isodense to hyperintense enhancement on T2-weighted imaging, with homogenous enhancement on postcontrast imaging [25, 26]. In addition to neuroimaging, the PCNSL Collaborative Group recommends ruling out non-CNS disease with full-body PET imaging and bone marrow biopsy. The confirmatory diagnostic test of choice is a stereotactic biopsy. Macroscopically, PCNSL is often identified as a well-circumscribed mass [27]. Microscopically, the tumor has highly proliferative tumor cells with an angiocentric pattern with centroblastic or immunoblastic tumor cells within and around the cerebral blood vessels [24]. The majority of PCNSL are DLBCL (90%) and less commonly Burkitt's lymphoma or T-cell lymphoma [6, 28]. Immunohistochemically, PCNSL cells stain positive for CD20 and Bcl-2 [24]. PCNSL cells are less frequently involved in translocations with *IGH*, *BCL6*, and *MYC* compared to systemic lymphomas [24].

While treatment of PCNSL has evolved in the past decade, many recommendations are derived from phase II clinical trials. In general, high-dose methotrexate (HD-MTX) and rituximab are recommended for initial induction agents due to improved overall response rates (ORR) and progression-free survival (PFS) [29]. Recently, the IELSG32 trial demonstrated that induction therapy with HD-MTX/leucovorin and rituximab with cytarabine improved the ORR (73 vs. 53%) and median PFS (20 months vs. 6 months) [30]. The multicenter cancer and leukemia group B study 50202 used rituximab, HD-MTX, and temozolomide followed by consolidation with high-dose etoposide and cytarabine with a reported ORR of 72% and PFS of 48 months, which is comparable to combination chemoradiation [28]. The Radiation Therapy Oncology Group 0227 trial (RTOG) used induction chemotherapy with rituximab, HD-MTX, and temozolomide followed by whole-brain radiation therapy (WBRT) and postirradiation temozolomide with an ORR of 86% and PFS of 90 months [31].

Both intrathecal chemotherapy (ITC) and surgical resection have been studied for the treatment of PCNSL, though routine use of either modality remains controversial. Despite the concern that CSF can harbor lymphoma cells and contribute to treatment failure or disease relapse [29], there has been no observed benefit of ITC on PFS or ORR in retrospective trials [32, 33]. In addition, surgical resection is not a recommended treatment modality due to the degree of tumor infiltration and risk of both postoperative neurologic damage and implantation metastasis associated with resection. Retrospective studies have not demonstrated a mortality benefit [23, 34], except for the German PCNSL study group 1 trial which reported improved OS in patients undergoing gross subtotal or total resection [35]. However, this advantage was lost when controlling for the total number of lesions [35]. Of patients with 4^th^ ventricular PCNSL, 35.3% of patients received ITC [8–11, 14, 16], and 52.9% of patients with 4^th^ ventricular PCNSL had gross subtotal or total resection [8, 11–17, 22]. These patients also received various combinations of chemotherapy and chemoradiation so whether either of these treatment modalities conferred a survival benefit in this select patient population is not known.

## 4. Conclusion

PCNSL arising from the 4^th^ ventricle is a rare occurrence that is seldom described in the literature. This case provides further evidence that PCNSL is often a malignancy of immunocompetent males. Due to its uncommon occurrence, it is unknown if commonly utilized treatment modalities that are employed in other anatomic variants of PCNSL will have similar impact on overall survival and progression-free survival. While surgical resection has been used in over half of 4^th^ ventricular PCNSL, it is unknown how this impacted OS or PFS. Further research is needed to determine the optimal treatment for the many variants of PCNSL, which may vary by location or tumor subtype.

## Figures and Tables

**Table 1:** Summary of findings in individuals with 4^th^ ventricular central nervous system lymphoma, including age, sex, immune status at presentation, initial symptoms, lymphoma subtype, treatment, and survival [7–22].

Author, year	Age/sex	Immune status	Symptoms	Lymphoma subtype	Treatment	Survival
Werneck et al., 1977 [7]^∗^	17/F	IC	Meningeal signs	PCNSL	Unknown	Postmortem diagnosis
Haegelen et al., 2001 [8]	33/F	IC	Headache, vertigo, and ataxia	High-grade BCL	Resection, chemoradiation, ITC, and aSCT	No recurrence at 7 months
Hill et al., 2009 [9]	69/M	IC	Vomiting, nausea, anorexia, and weight loss	DLBCL	Chemotherapy, ITC	No recurrence at 3 months
Brar et al., 2012 [10] *α*	65/F	IC	Headache, nausea, and vomiting	High-grade BCL	Chemotherapy, ITC	No recurrence at 2 months
Bokhari et al., 2013 [11]	50/M	IC	Vomiting, nausea, headache, and confusion	DLBCL	Resection, chemoradiation, and ITC	No recurrence at 18 months
Rao et al., 2013 [12]	59/M	IC	Vomiting, nausea, vertigo, tremors of upper limbs and hands, and ataxia	DLBCL	Resection, chemotherapy	No recurrence at 8 months
Liao et al., 2014 [13]	77/M	IC	Vertigo, nausea, vomiting, and ataxia	DLBCL	Resection	No recurrence at 9 months
Fabiano et al., 2014 [14]	60/F	IC	Diplopia	DLBCL	Resection, chemoradiation, and ITC	No recurrence at 6 months
Grossman et al., 2014 [15]	66/M	IC	Ataxia, diplopia	PCNSL	Resection, unknown if further therapy	Unknown
Alabdulsalam et al., 2014 [16]	18/M	IC	Ataxia, cranial nerves IV, VII, IX, and X palsies	Burkitt	Resection, chemotherapy, and ITC	No recurrence at 18 months
Hsu et al., 2015 [17]	61/M	IC	Headache, dizziness, and ataxia	DLBCL	Resection, chemotherapy	No recurrence at 3 months
Suri et al., 2015 [18] *β*	15/M	IC	Headache, nausea, vomiting, and generalized tonic clonic seizure	DLBCL	Unknown	Unknown
Zhu et al., 2015 [19] ¥	66/M	IC	Headache, dizziness, diplopia, and cranial nerve VI, VII palsy	DLBCL	Chemotherapy	No recurrence at 6 months
Cellina et al., 2015 [20] €	65/M	IC	Weight loss, headache, diplopia, and ataxia	DLBCL	Chemotherapy	No recurrence at 2 weeks
Liu et al., 2016 [21]	6/M	IC	Headache	Burkitt	Unknown	Unknown
Yi et al., 2017 [22]	61/M	IC	Headache, confusion, ataxia, and urinary incontinence	DLBCL	Resection, chemoradiation	No recurrence at 20 months
Current case ψ	65/M	IC	Diplopia, vertigo, nausea, vomiting, weight loss, ataxia, and simple partial seizures	DLBCL	Chemoradiation, ITC	No recurrence at 8 months

^∗^: lymphoma in the 4^th^ ventricle and meninges. *α*: lymphoma in the 4^th^ ventricle and right lateral ventricle. *β*: lymphoma in the 4^th^ ventricle and bilateral ventricles. ¥: lymphoma in the 4^th^ ventricle and right lateral ventricle. €: lymphoma in the 4^th^ ventricle and hypothalamus. Ψ: lymphoma in the 4^th^ ventricle and pituitary gland. F: female; M: male; IC: immunocompetent; DLBCL: diffuse large B-cell lymphoma; BCL: B-cell lymphoma; ITC: intrathecal chemotherapy; aSCT: autologous stem cell transplant.

## Abbreviations

MRI: Magnetic resonance imaging
DLBCL: Diffuse large B-cell lymphoma
PCNSL: Primary CNS lymphoma
NHL: Non-Hodgkin lymphoma
LDH: Lactate dehydrogenase
CT: Computed tomography
PET: Positron emission tomography
Hyper-CVAD: Cyclophosphamide, vincristine, doxorubicin, and dexamethasone
CSF: Cerebrospinal fluid
ITC: Intrathecal chemotherapy
PFS: Progression-free survival
ORR: Overall response rate
OS: Overall survival
MRV: Magnetic resonance venography.
